# TRPM3 in Brain (Patho)Physiology

**DOI:** 10.3389/fcell.2021.635659

**Published:** 2021-02-26

**Authors:** Katharina Held, Balázs István Tóth

**Affiliations:** ^1^Laboratory of Endometrium, Endometriosis and Reproductive Medicine, Department of Development and Regeneration, KU Leuven, Leuven, Belgium; ^2^Laboratory of Ion Channel Research, Department of Cellular and Molecular Medicine and VIB-KU Leuven Center for Brain and Disease Research, KU Leuven, Leuven, Belgium; ^3^Laboratory of Cellular and Molecular Physiology, Department of Physiology, Faculty of Medicine, University of Debrecen, Debrecen, Hungary

**Keywords:** ion channels, channelopathies, transient receptor potential melastatin 3 channel, pregnenolone sulfate, brain, neurological disorders, gating pore current

## Abstract

Already for centuries, humankind is driven to understand the physiological and pathological mechanisms that occur in our brains. Today, we know that ion channels play an essential role in the regulation of neural processes and control many functions of the central nervous system. Ion channels present a diverse group of membrane-spanning proteins that allow ions to penetrate the insulating cell membrane upon opening of their channel pores. This regulated ion permeation results in different electrical and chemical signals that are necessary to maintain physiological excitatory and inhibitory processes in the brain. Therefore, it is no surprise that disturbances in the functions of cerebral ion channels can result in a plethora of neurological disorders, which present a tremendous health care burden for our current society. The identification of ion channel-related brain disorders also fuel the research into the roles of ion channel proteins in various brain states. In the last decade, mounting evidence has been collected that indicates a pivotal role for transient receptor potential (TRP) ion channels in the development and various physiological functions of the central nervous system. For instance, TRP channels modulate neurite growth, synaptic plasticity and integration, and are required for neuronal survival. Moreover, TRP channels are involved in numerous neurological disorders. TRPM3 belongs to the melastatin subfamily of TRP channels and represents a non-selective cation channel that can be activated by several different stimuli, including the neurosteroid pregnenolone sulfate, osmotic pressures and heat. The channel is best known as a peripheral nociceptive ion channel that participates in heat sensation. However, recent research identifies TRPM3 as an emerging new player in the brain. In this review, we summarize the available data regarding the roles of TRPM3 in the brain, and correlate these data with the neuropathological processes in which this ion channel may be involved.

## Introduction

The brain forms the control center of our body and is responsible for processing tremendous amounts of data to monitor and regulate our bodily functions at day and night. Such a precise control needs to be fast and accurate and requires highly sophisticated information processing. Our body accomplishes this task via neuronal cells, which form a complex connectome within the nervous system (Purves, 2004). Within the nervous system, information is delivered and processed in form of electrical signals and synaptic events. Electrical signals occur, propagate and get transduced by neurons due to a diverse set of ion channels present in the membranes of the nerve cells (Hille, 2001). Ion channel proteins allow the permeation of ions over the otherwise ion-impermeable cell membrane, which results in a potential difference between the extracellular space and the intracellular side of the cell known as the membrane potential. Practically, changes in membrane potential serve as the information bearing electrical signals and are strictly controlled by ion channels (Hille, 2001; Purves, 2004). Therefore, genetic or acquired alterations in function of ion channels can result in severe disturbances of the electrical signaling in our nervous system causing several neurological or psychiatric diseases. For this reason, ion channels are primary targets for pharmacological interventions to treat such diseases (Kullmann, 2002; Kumar et al., 2016).

**Figure 1:**
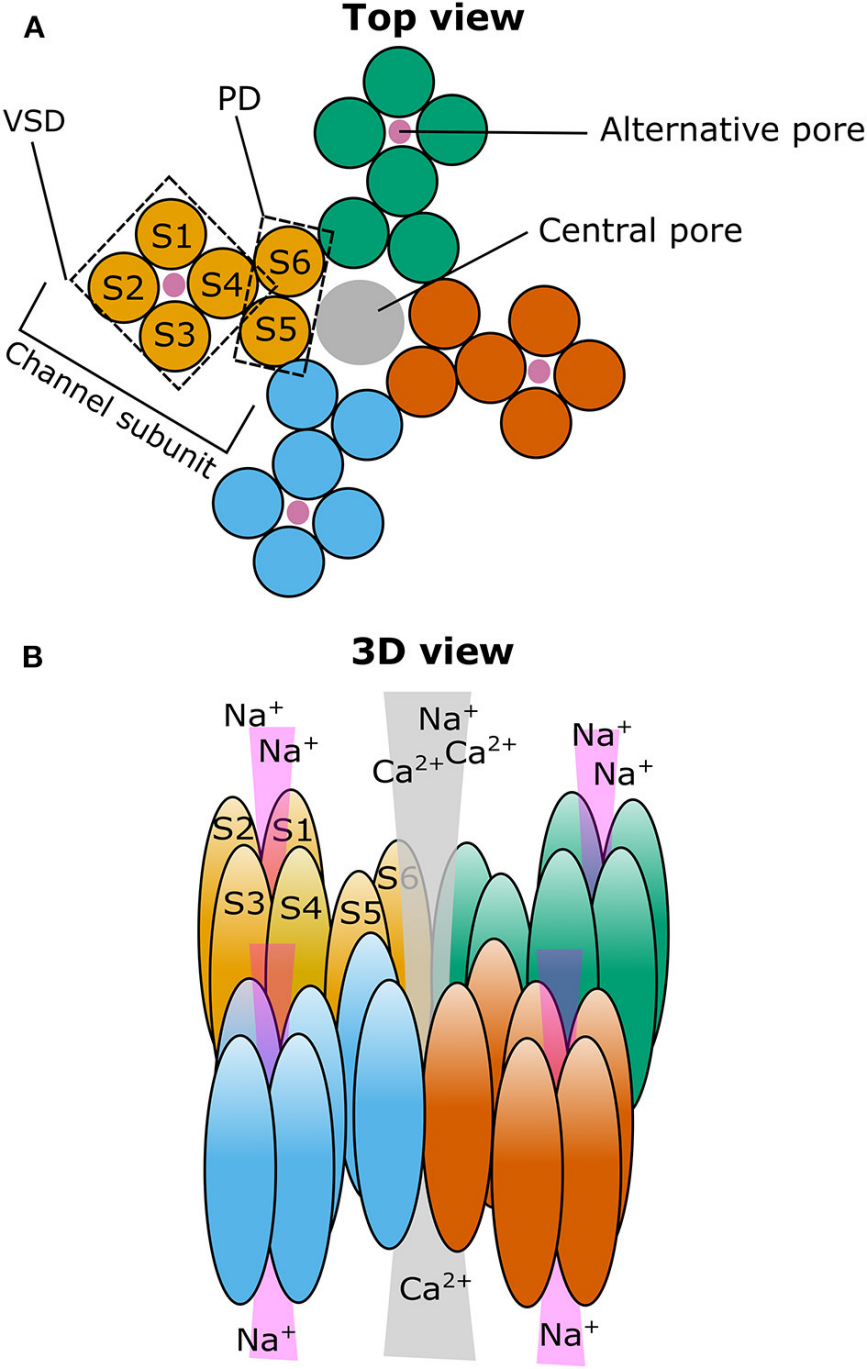
Cartoon illustrating the putative localization of the central and alternative ion pore in TRPM3. **(A)** Top view on a representation of the four TRPM3 channel subunits (individually colored) that come together to form a functional ion channel. The voltage-sensing domain (VSD) and the pore domain (PD) are indicated in one of the subunits as well as the path of the central pore (gray circle) and the putative path(s) for the alternative ion pore(s) (four magenta circles). **(B)** Same as in **(A)** but in a three-dimensional representation looking from the diagonal top on TRPM3. S1–6: Transmembrane segment 1–6.

Among the hundreds of ion channels expressed in the human brain, Transient Receptor Potential (TRP) channels form a diverse group of poly-modally activated cation channels and they are generally considered as molecular sensors of external and internal stimuli (Clapham, 2003; Voets et al., 2005; Nilius, 2012). In total, 28 different TRP channels exist in mammals, which are divided into seven subfamilies according to their sequence homology. Functionally active TRP channels are composed of four individual subunits (Clapham et al., 2005) as recently evidenced by high resolution structural models (Liao et al., 2013; Yin et al., 2018; Cao, 2020; Huang et al., 2020) (Figure 1). Once activated, TRP channels allow the influx of cations into the intracellular space resulting in the depolarization of the plasma membrane and possible subsequent modulation of voltage-gated ion channels (Clapham et al., 2005; Gees et al., 2010; Wu et al., 2010; Nilius and Szallasi, 2014). Importantly, most TRP channels possess a substantial permeability for calcium, which is a vital signaling molecule throughout several cellular and molecular processes and therefore, plays a leading role in brain homeostasis and excitability (McBurney and Neering, 1987; Zündorf and Reiser, 2011). It is generally believed that TRP channels can play a substantial role in fine-tuning the membrane potential and neuronal excitability (Sawamura et al., 2017).

Some members of the TRP channel family are functionally expressed in various brain regions and their involvement in diverse physiological and pathological processes of the brain has already been described (Moran et al., 2004; Nilius, 2012; Reboreda, 2012; Sawamura et al., 2017). Among them, the TRP **c**anonical (TRPC) channel subfamily presents probably the most-studied TRP channel subfamily in the brain (Sawamura et al., 2017). TRPCs were reported in various brain regions (Riccio et al., 2002; Huang et al., 2011), where they were implicated in excitatory post-synaptic conduction (Kim et al., 2003), neuronal cell death and survival (Jia et al., 2007; Narayanan et al., 2008), and dendritic growth and spine formation (Amaral and Pozzo-Miller, 2007; Tai et al., 2008), among other things. Another well-studied TRP channel in the brain is TRP **v**anilloid 1 (TRPV1), which is of importance in descending modulatory pain pathways and seems to play an extended role in other brain functions, by enhancing glutamatergic neurotransmission (Martins et al., 2014).

The TRP **m**elastatin (TRPM) channel subfamily represents an especially heterogeneous group within the TRP superfamily and includes eight members in total (TRPM1-8). Most of them are non-selective calcium-permeable cation channels. As an exception, TRPM4 and TRPM5 are calcium-impermeable channels activated by intracellular calcium. As such, all TRPM family members can evoke calcium increases in cells, either by conducting calcium ions through their pores or by regulating the membrane potential, thereby activating/modulating calcium channels or transporters (Clapham et al., 2005; Wu et al., 2010; Huang et al., 2020). Moreover, three members (TRPM2, TRPM6 and TRPM7) are so-called “chanzymes,” bearing an enzyme (kinase or hydrolase) domain in addition to their ion channel pore (Nilius and Owsianik, 2011; Huang et al., 2020). Furthermore, several TRPM channels are also thermosensitive, covering a wide range of physiological temperatures (TRPM2, TRPM3, TRPM4, TRPM5 and TRPM8) (Talavera et al., 2005; Dhaka et al., 2007; Vriens et al., 2011; Tan and McNaughton, 2018; Vandewauw et al., 2018). The diverse properties of the TRPM family explain their involvement in a multitude of biological processes. They are regulating sensory processes, including vision (TRPM1) (Morgans et al., 2009), taste (TRPM5) (Pérez et al., 2002; Talavera et al., 2005), temperature sensation and nociception (TRPM2, TRPM3, TRPM8) (Dhaka et al., 2007; Voets et al., 2007; Vriens et al., 2011; Held et al., 2015b; Tan and McNaughton, 2018; Vandewauw et al., 2018). Moreover, they play important roles in ion homeostasis (Mg^2+^ uptake and reabsorption by TRPM6 and TRPM7) (Schlingmann et al., 2007), and modulate secretory processes (TRPM2, TRPM3, TRPM4, TRPM5) in various cells all over the body (Cheng et al., 2007; Wagner et al., 2008; Brixel et al., 2010; Mathar et al., 2010; Uchida et al., 2011; Held et al., 2015a; Philippaert et al., 2017).

TRPM2 represents the best characterized member in the brain, where it exhibits an ubiquitous expression pattern (Allen Mouse Brain Atlas, 2004; Fonfria et al., 2006; Lein et al., 2007). It is intensively studied with regards to its role in the response to oxidative stress, which occurs during aging and neurodegenerative diseases (Sita et al., 2018). Recently, TRPM2 was also described as a hypothalamic heat sensor involved in central thermoregulation (Song et al., 2016). Furthermore, TRPM7 is widely expressed in the brain, and was shown to be involved in cell growth (Turlova et al., 2016) and cell death after ischemic and hypoxic brain injuries (Aarts et al., 2003; Sun et al., 2009; Chen et al., 2015; Sun, 2017). TRPM4 was also reported to be involved in cognitive functions, as well as in (patho)physiological processes, such as hippocampal plasticity (Menigoz et al., 2016; Bovet-Carmona et al., 2018) or development of trauma induced brain edema (Gerzanich et al., 2019; Woo et al., 2020).

TRPM3, a less studied member of the melastatin subfamily (Grimm et al., 2003; Lee et al., 2003), recently came into the focus of attention due to its involvement in human brain pathologies. In fact, TRPM3 is best characterized in the peripheral nervous system, where it functions as a noxious heat sensor in somatosensory neurons (Vriens et al., 2011; Vriens and Voets, 2018). Although several groups had indicated an abundant expression of TRPM3 in the brain in the past (Oberwinkler and Philipp, 2014), only few studies investigated its roles in the central nervous system, showing its functional presence in cerebellar Purkinje cells (Zamudio-Bulcock et al., 2011) and oligodendrocytes (Hoffmann et al., 2010). The more surprising were two recent publications that reported *de novo* mutations in TRPM3 as the cause of developmental and epileptic encephalopathies (DEEs) in a total of nine patients (Dyment et al., 2019; de Sainte Agathe et al., 2020). These findings motivated further functional studies on these mutant channels (Van Hoeymissen et al., 2020; Zhao et al., 2020).

In this review, we summarize our current knowledge and knowledge gaps related to TRPM3, focusing on channel properties that are relevant to understand its role in brain function and pathology.

## Ion Channel Properties and Functions of TRPM3—Lessons From the Peripheral Nervous System and Non-neural Tissues

### General Properties of TRPM3 Splice Variants

TRPM3 was identified <20 years ago as a functional ion channel-forming TRP protein (Grimm et al., 2003; Lee et al., 2003). In 2007, it was even labeled as an enigmatic channel (Oberwinkler, 2007), based on the fact that the *Trpm3* gene appears to encode the highest number of channel isoforms reported within the TRP family (Oberwinkler and Philipp, 2014; Shiels, 2020). The different isoforms arise from alternative splicing at the N terminal part of the channel and from alternative splicing of exons spread all over the gene. The mouse isoforms are classified into TRPM3α and TRPM3β groups depending on the start exon. TRPM3α isoforms start with exon 1 and do not express exon 2, while TRPM3β isoforms start with exon 2 (Oberwinkler et al., 2005; Oberwinkler, 2007; Oberwinkler and Philipp, 2014). Recently, the novel splice variants, TRPM3γ2 and TRPM3γ3 were identified, which start with exon2 and have a truncation in exon 28 (Uchida et al., 2019). As in mouse, several splice variants exist in humans, labeled as TRPM3a-f following their relative abundance (Lee et al., 2003). Even more transcript variants and predicted protein sequences are deposited in the public NCBI reference sequence (RefSeq) database (Shiels, 2020). The splicing pattern seems to be well-conserved between species (Oberwinkler and Phillipp, 2007; Oberwinkler and Philipp, 2014). However, most of the reported isoforms are not yet functionally characterized and their roles are largely unknown. Whether they form functional cation channels seems to depend on a specific *r*egion *i*ndispensable for *c*hannel *f* unctions (ICFR), which was identified to be essential for the channel formation. Isoforms lacking ICFR, like TRPM3α7, do not form functional ion channels, probably due to a disturbed tetrameric channel complex formation and a decreased plasma membrane expression. Therefore, when co-expressed with the functional TRPM3α2, TRPM3α7 acts as a dominant negative regulator of the channel activity (Frühwald et al., 2012). Although TRPM3α7 transcripts were detected and estimated to form about 15% of the total TRPM3 transcripts in the brain (Frühwald et al., 2012), its impact on brain-specific function is yet to be discovered.

Upon cloning, both human and mouse TRPM3 were identified as Ca^2+^ entry channels (Grimm et al., 2003; Lee et al., 2003), but their permeability and functional features can vary tremendously between different isoforms. At this point, only a few of the TRPM3 isoforms have been functionally characterized in detail, with the most comprehensive analyses comparing the isoforms TRPM3α1 and TRPM3α2 (Oberwinkler et al., 2005; Held et al., 2020), which differ only in the presence (TRPM3α1) or absence (TRPM3α2) of a 12 amino acid-long sequence insertion into the pore-forming loop. This seemingly minor difference results in a massive change in their biophysical characteristics, which dramatically affects their permeability. Homotetrameric ion channels formed by TRPM3α2 subunits are permeable for Ca^2+^, Mg^2+^, and even for Zn^2+^ and other divalent cations. In contrast, TRPM3α1 displays strongly reduced permeability for divalent cations and high selectivity toward monovalent cations (Oberwinkler et al., 2005; Wagner et al., 2010). Similar to other TRP channels, both isoforms are strongly inhibited by intracellular Mg^2+^ and show reduced ionic currents in the presence of extracellular divalent cations. The permeability of TRPM3α2, but not TRPM3α1, is also markedly reduced by extracellular monovalent cations (Oberwinkler et al., 2005). In addition to differences in selectivity, there are also marked differences in the pharmacological properties of the short and long pore loop variants. For instance, the long pore loop variant TRPM3α1 is insensitive to pregnenolone sulfate (PregS), a well-characterized agonist of the short pore loop variants TRPM3α2-6, and the isoforms show different sensitivities to several other agonists and antagonists, as well (Held et al., 2020), as detailed later in this review. The recently described isoforms TRPM3γ2 and TRPM3γ3 (Uchida et al., 2019) exhibit biophysical and pharmacological characteristics that appear very similar to those of TRPM3α2, although they show a generally decreased channel activity.

The high diversity amongst TRPM3 isoforms raises the physiologically highly relevant question to what extent do they contribute to the formation of the native TRPM3 channels in the various tissues. Although the answer is uncertain, the data discussed below suggest that most native TRPM3 channels exhibit biophysical and pharmacological properties that are similar to those of TRPM3α2. Indeed, publicly available transcriptome data from mouse tissues demonstrate that the short pore loop isoforms generally dominate, although to varying degrees in different tissues (Held et al., 2020). The transcripts of the recently described γ isoforms are also highly expressed in dorsal root ganglia (DRGs), but their expression in the brain is not known. Moreover, these γ isoforms were suggested not to interact with TRPM3α2 and not to alter pharmacological properties of TRPM3α2 when co-expressed in a recombinant system (Uchida et al., 2019).

The most abundant and characterized human isoform TRPM3a also shares the short pore loop with TRPM3α2 (Oberwinkler and Phillipp, 2007) and both their pharmacological properties and functional features are very similar (Badheka et al., 2015, 2017; Held et al., 2015a). Native human TRPM3 expressed in the sensory ganglia also seems to be functionally similar to the mouse TRPM3α2 channel (Vangeel et al., 2020). Cumulatively, these data indicate that the mouse channel, especially the TRPM3α2 variant, is a highly relevant model to study the function of (native) human TRPM3. Therefore, in the following parts we use the term TRPM3 when referring to the TRPM3α2 variant or to native channels, and specify distinct other variants when relevant.

It is important to mention that, besides the various isoforms, the *Trpm3* gene also codes a microRNA, miR-204, in intron 9 in both humans and mice. It is often co-expressed with the TRPM3 ion channel and should be considered in certain situations when investigating TRPM3 functions, especially when analyzing gene-deleted animal models (Oberwinkler and Philipp, 2014; Shiels, 2020). TRPM3 and miR-204 are co-expressed in pancreatic beta cells and affect insulin production and secretion (Wagner et al., 2008; Thiel et al., 2013; Xu et al., 2013). They are also highly co-expressed in several cells of the eye and seem to be regulated by the same promoter and transcription factors, including paired-box 6 transcription factor (Pax6) and microphthalmia/melanogenesis-associated transcription factor (MITF). These results suggest a possible synergism between their function in eye development and the onset of some ocular diseases (Karali et al., 2007; Adijanto et al., 2012; Xie et al., 2014; Shiels, 2020). Interestingly, TRPM1, the closest relative of TRPM3, also hosts a microRNA, miR-211, which belongs to the same microRNA family as miR-204 and also plays a significant role in the eye (Shiels, 2020). Moreover, in clear cell renal cell carcinoma, TRPM3 and miR-204 were found to play an antagonistic role in the control of oncogenic autophagy (Hall et al., 2014).

### (Patho)physiological Roles of TRPM3 in the Periphery

TRPM3 was originally described as a constitutively active Ca^2+^ entry channel in the plasma membrane, which can contribute to store-operated Ca^2+^ entry in certain conditions (Lee et al., 2003), although later studies suggested that store depletion is not significant in regulating TRPM3 activity (Grimm et al., 2003, 2005; Oberwinkler and Phillipp, 2007). The activity of recombinant TRPM3 expressed in HEK293 cells can be stimulated by hypotonic solutions (Grimm et al., 2003; Held et al., 2020), although it remains unclear whether TRPM3 is directly activated by hypotonicity-related membrane stretching or the hypoosmotic environment activates the channel indirectly (Oberwinkler and Phillipp, 2007). TRPM3 was reported to mediate the effects of hypotonic stress in different context: decreased serum osmolarity stimulated the constriction of ductus arteriosus via activation of TRPM3 (Aoki et al., 2014) and hypotonic stress-induced upregulation of the receptor activator of nuclear factor kappa-B ligand (RANKL) and the nuclear factor of activated T cells type c1 (NFATc1) was mediated by both TRPM3 and TRPV4 in periodontal ligament cells and osteoblasts (Son et al., 2015, 2018). These results suggest that TRPM3 can contribute to the mechanical stress-induced bone remodeling. Oppositely, inhibition of TRPM3 by hypertonic solutions may contribute to hypertonicity-induced gene expression in ciliated renal epithelial cells. However, conclusions of this study may be regarded with some caution, as the expression of TRPM3 was confirmed with antibodies and at RNA level, but TRPM3-mediated currents were not reported. Furthermore, the pharmacological effect of the TRPM3 agonist PregS on the hypertonic (500 mosM NaCl) solution-induced gene expression was only very partial, although it could be reversed by the TRPM3 antagonist isosakuranetin (Siroky et al., 2017). Another study, using pharmacological tools, suggested the presence of TRPP2-TRPM3 heteromeric channels in renal primary cilia (Kleene et al., 2019). In contrast to the most widespread variant, the long pore loop isoform TRPM3α1 is not sensitive to hypotonic clues (Held et al., 2020).

TRPM3 is also expressed in vascular smooth muscle cells, where its activation evoked smooth muscle contraction resulting in vasoconstriction, and inhibited IL-6 secretion (Naylor et al., 2010). However, activation of TRPM3 in the perivascular nerves resulted in vasodilation via release of vasoactive substances from perivascular nerve endings (Alonso-Carbajo et al., 2019). Stimulation of TRPM3 also inhibited secretory activity in fibroblast-like synoviocytes from rheumatoid arthritis patients, as TRPM3 agonist inhibited hyaloronan release (Ciurtin et al., 2010). The pharmacological activation of TRPM3 channels expressed in pancreatic beta cells induced insulin secretion (Wagner et al., 2008; Colsoul et al., 2011; Becker et al., 2020). Upon its activation, TRPM3 stimulated the opening of voltage-gated Ca^2+^ channels and initiated complex signaling pathways, upregulating the expression of different transcription factors (Mayer et al., 2011; Müller et al., 2011; Thiel et al., 2013; Becker et al., 2020). The opening of TRPM3 also serves as a regulated Zn^2+^ entry pathway in beta cells (Wagner et al., 2010), where zinc plays a relevant role in the biosynthesis and storage of insulin (Dunn, 2005). Interestingly, extracellular zinc entering via TRPM3 or voltage-gated Ca^2+^ channels can inhibit gene expression initiated by Ca^2+^ entry via the same channels. Therefore, the zinc release accompanying insulin release is hypothesized to act as a negative feedback on exocytosis (Loviscach et al., 2020). Zinc plays an important role in the central nervous system as well, by regulating excitability of ion channels and it can be released together with glutamate (Frederickson et al., 2005; Sensi et al., 2009), but until today, the role of TRPM3 in zinc-related signaling in the brain remains elusive.

TRPM3 is intensely studied in the somatosensory neurons of DRGs and trigeminal ganglia. In 2011, it was introduced as a new member of the thermosensitive TRP channels activated by warming. Elevation of temperature from room temperature to 33°C potentiates agonist induced TRPM3 activation, but its sensitivity toward warming is more dominant in the noxious heat range (>42°C) (Vriens et al., 2011, 2014b; Voets, 2012; Held et al., 2015b). Interestingly, heat sensitivity is completely lost in the long pore loop variant TRPM3α1 (Held et al., 2020). Compared to the well-characterized heat sensor TRPV1, the current–temperature relationship curve of TRPM3 is shifted slightly toward higher temperatures and its temperature-dependent increase in open probability is less steep (Vriens et al., 2011, 2014b; Voets, 2012; Held et al., 2015b). In good accordance with its thermosensitivity and expression by small-sized somatosensory neurons, TRPM3 plays a role in noxious heat sensation together with TRPV1 and TRPA1 (Vriens et al., 2011; Vandewauw et al., 2018; Vriens and Voets, 2018, 2019). TRPM3 activation results in neuropeptide release from the sensory terminals (Held et al., 2015a) and the channel is sensitized by inflammatory conditions, which may contribute to inflammatory hyperalgesia (Vriens et al., 2011; Mulier et al., 2020). In contrast to TRPV1, TRPM3 does not to appear to play a role in central thermoregulation and neither agonists nor antagonists induce noticeable changes in core body temperature (Vriens et al., 2011; Straub et al., 2013a). It selectively mediates pain, and is not involved in itch evoked by pruritic mediators such as histamine or serotonin (Kelemen et al., 2021), which are known to signal via TRPV1 (Shim et al., 2007) and TRPA1 (Morita et al., 2015), respectively.

In addition to somatosensory afferents, TRPM3 is also functional in the vagal afferents of the nodose ganglion (Staaf et al., 2010; Fenwick et al., 2014; Wu et al., 2014; Ragozzino et al., 2020). These channels contribute to basal and temperature-driven spontaneous glutamate release from the central terminals in the nucleus tractus solitarii, while not affecting the synchronous or asynchronous glutamate release (Ragozzino et al., 2020).

### Regulation of the Channel Activity and Pharmacology of TRPM3

#### Intrinsic Regulation by Signaling Molecules

Until today, only a few intracellular signaling molecules were reported to interact with TRPM3. Like other TRP channels, TRPM3 activity is reduced by intracellular Mg^2+^ (Oberwinkler et al., 2005) and Ca^2+^ (Przibilla et al., 2018), and potentiated by phosphatidylinositol 4,5-bisphosphate [PtdIns(4,5)P_2_] and other phosphoinositides, among which PtdIns(3,4,5)P_3_ was found to be the most effective (Badheka and Rohacs, 2015; Badheka et al., 2015; Tóth et al., 2015, 2016; Uchida et al., 2016). Multiple Ca^2+^-calmodulin binding sites on the N-terminus were identified, which may interact with PtdIns(4,5)P_2_, and S100A protein (Holakovska et al., 2012; Holendova et al., 2012; Przibilla et al., 2018). In good accordance, signaling pathways decreasing endogenous PtdIns(4,5)P_2_, like phospholipase C activation evoked by M1 or M3 muscarinic acetylcholine receptors, inhibited both recombinant and native TRPM3 (Badheka et al., 2015; Tóth et al., 2015). G_βγ_ subunits of trimeric G-proteins were also shown to be negatively coupled to TRPM3 activity, which underlies inhibition of TRPM3 upon stimulation of several G-protein-coupled receptors, including G_q_-coupled M1 muscarinic acetylcholine, B2 bradykinin receptors and G_i_-coupled M2 muscarinic acetylcholine, D2 dopamine, GABA_B_, neuropeptide Y, μ-opioid receptors and G_s_-coupled EP-2 prostaglandin, and A2B adenosine receptors or receptors of somatostatin (Badheka et al., 2017; Dembla et al., 2017; Quallo et al., 2017; Alkhatib et al., 2019). A 10-amino-acid-long domain in TRPM3 was identified that interacted with G_βγ_ proteins. This domain is encoded in an alternatively spliced exon, and is absent in the TRPM3α4 and TRPM3α5 variants, rendering them insensitive to μ-opioid receptor activation or overexpressed Gβ_1_γ_2_ subunits. X-ray crystallographic analysis of the corresponding peptide bound to Gβ_1_γ_2_ revealed that this domain of TRPM3 interacts exclusively with the Gβ_1_ (and not Gγ_2_) subunit, and amino acids on both the TRPM3 peptide and the Gβ_1_-proteins that mediate the interaction were identified. Interestingly, the interacting residues in Gβ_1_ only partially overlap with those involved in the inhibitory interaction with G-protein-coupled inward rectifier K^+^ (GIRK) channels (Behrendt et al., 2020). These results suggest that pharmacological targeting of TRPM3 may have a great potential to influence several signaling pathways relevant in various brain functions and in peripheral pain sensation.

#### TRPM3 Is a Steroid Regulated Channel

The first chemical activator of TRPM3 to be identified was D-erythro sphingosine, which was known from earlier studies to inhibit other ion channels. Two structural analogs, dihydro-D-erythro-sphingosine and N,N-dimethyl-D-erythro-sphingosine, although less effectively, also activated TRPM3, while neither ceramide and 1-sphingosine-phosphate (other significant signaling lipids of the sphingolipid pathway), nor arachidonic acid, anandamide, linoleic acid, linolenic acid, and diacylglycerol analogs affected the channel (Grimm et al., 2005). However, the TRPM3 specificity of D-erythro sphingosine was questioned later (Wagner et al., 2008). The L-type Ca^2+^ channel blocker nifedipine also activated TRPM3 in a reversible way (Wagner et al., 2008), but the long pore loop variant TRPM3α1 was found to be insensitive for nifedipine (Held et al., 2020).

The best characterized and most widely used endogenous TRPM3 agonist is the steroid compound PregS. It activates the channel by shifting its current-voltage activation curve toward more negative membrane potentials and also potentiates temperature-induced activation (Wagner et al., 2008; Vriens et al., 2011; Held et al., 2018). These mechanisms of action are typical features of the agonist-evoked activation of thermosensitive TRP channels in general (Voets et al., 2004). Although PregS activated TRPM3 only in supraphysiological concentrations in most of the experiments carried out at room temperature, a marked activation was evoked by only 100 nM PregS at 37°C, which is in the range of the physiological plasma concentrations (Vriens et al., 2011). Therefore, PregS can be considered as an endogenous activator of TRPM3, even in physiological circumstances. Due to the presence of the negatively charged sulfate group, pregS is a quite lipophobic substance and it behaves as a membrane-impermeable ligand that activates the channel only if applied to the extracellular side (Wagner et al., 2008). This finding suggests that the steroid-binding pocket of TRPM3 is located on the extracellular surface. The steroid (PregS) sensitivity is conserved in the short pore loop (TRPM3α2-α6) variants but completely lost in the long pore loop (TRPM3α1) variant, arguing for the importance of the pore region in the steroid activation of the channel (Held et al., 2020). Analysis of the structure-activity relationship of the steroid ligands revealed that the natural PregS is more effective than its enantiomer and the position and orientation of the sulfate group is also very important to preserve TRPM3 activation (Majeed et al., 2010; Drews et al., 2014). A few other, structurally similar steroid compounds [pregnenolone, dehydroepiandrosterone (DHEA) and DHEA-sulfate] also evoked a moderate activation of TRPM3 (Wagner et al., 2008).

Other steroids were also tested for activity toward the channel. In contrast to PregS, dihydrotestosterone, 17β-estradiol, and progesterone and its metabolites inhibited TRPM3 activation. The inhibitory effect of progesterone was independent of the used TRPM3 activator, whereas dihydrotestosterone behaved as competitive antagonist of PregS (Majeed et al., 2012).

#### Opening of a Non-canonical Pore and Its Significance

We found that activation by PregS is strongly potentiated by the co-application of the antifungal clotrimazole or its structural analogs TRAM34, senicapoc, and tamoxifen. Importantly, clotrimazole did not only potentiate the outwardly rectifying PregS evoked currents flowing through the well-established central pore of the channel, but also evoked monovalent-selective inwardly rectifying currents at negative membrane potentials (Vriens et al., 2014a). The biophysical characteristics of these currents resemble the so-called omega currents or gating pore currents described earlier in mutated voltage-gated Na^+^ and K^+^ channels (Sokolov et al., 2005, 2007; Tombola et al., 2005, 2007). The existence of an alternative ion permeation pathway conducting “omega-like” currents in TRPM3 is supported by several lines of evidence. Among others, the voltage sensitivity and permeability of this alternative ion permeation pathway strikingly differs from the main pore, and, in contrast to the main pore, it is resistant to Ca^2+^-induced desensitization, as well as to classical pore blockers like La^3+^. Importantly, mutations in the pore domain disrupting the channel's permeability did not affect the alternative pore current, but several mutations generated in the voltage-sensing domain affected the gating pore current, underlining the fundamental role of the voltage sensor domain in forming the non-canonical pore (Figure 1). Furthermore, introducing an arginine residue (Trp982Arg) into the S4 segment of the voltage sensor domain prevented PregS and clotrimazole from activating the alternative pore current without inhibiting the main pore-related conductance (Vriens et al., 2014a; Held et al., 2018). Indeed, arginine residues are essential components of the S4 segment of voltage-gated ion channels, and eliminating these positively charged residues results in the appearance of the gating pore current in the above mentioned mutated K^+^ and Na^+^ channels (Sokolov et al., 2005; Tombola et al., 2005; Held et al., 2016). Based on our best knowledge, until today, the opening of non-canonical pores was revealed only in three naturally occurring wild type ion channels: a flatworm K_v_3 channel (N.at-K_v_3.2), the H_v_ proton channel, which even does not possess a classical pore domain (Ramsey et al., 2006, 2010; Sasaki et al., 2006; Okamura et al., 2015), and the mammalian TRPM3 (Vriens et al., 2014a). Alignment of the S4 segment of these channels to other voltage-gated ion channels reveals that some of the arginine residues are substituted by uncharged or negatively charged amino acid residues, thereby further highlighting that the loss of positively charged residues from the S4 plays a crucial role in the appearance of an alternative ion permeation pathway [For a comparative review about non-canonical pores in ion channels, we refer to our recent work (Held et al., 2016)]. Recently, we also identified CIM0216 as the currently available most potent and highly effective exogenous activator of TRPM3. Application of CIM0216 alone (i.e., without any other agonist) results in the simultaneous opening of both the classical and the non-canonical pore of the channel (Held et al., 2015a). However, until now, no endogenous ligand was identified that opens this alternative permeation pathway through TRPM3. Interestingly, clotrimazole applied on its own activated the PregS-insensitive long pore loop variant TRPM3α1, which resulted in a linear *I-V* relationship, including large inward currents at negative membrane potentials. The clotrimazole-evoked currents were found to be largely resistant to the classical pore blocker La^3+^ and to Ca^2+^ desensitization, suggesting a potential contribution of the non-canonical pore (Held et al., 2020).

Although, the physiological circumstances that may open the non-canonical pore of TRPM3 remain elusive, it may have important pathological significance. Compared to the exclusive activation of the main pore conductance, the additional opening of the non-canonical pore in TRPM3 increases the discharge rate of somatosensory neurons and exacerbates TRPM3-mediated nociception (Vriens et al., 2014a; Held et al., 2015a). Mutations in the voltage-sensing domain of Na_v_ and Ca_v_ channels that result in the appearance of ion conducting non-canonical pores are known to cause muscle (periodic paralysis) and heart diseases (Mixed Arrhythmias and Dilated Cardiomyopathy). Some mutations in the voltage sensor of K_v_7.2 and K_v_7.3 subunits also result in the development of non-canonical pore currents. These currents can contribute to the hyperexcitability of neurons and are associated with benign familial neonatal seizures. Recently, two mutations in TRPM3 were found in patients with developmental and epileptic encephalopathies (DEE) (Dyment et al., 2019; de Sainte Agathe et al., 2020). Notably, the mutations were characterized as gain of function mutations (Van Hoeymissen et al., 2020; Zhao et al., 2020), and in one of them the natural ligand PregS activated the gating pore currents (Van Hoeymissen et al., 2020).

#### Antagonists of TRPM3

Only few blockers of TRPM3 have been described. As nifedipine was recognized as an agonist of the channel, other dihydropyridines were also tested and found to inhibit TRPM3 (Drews et al., 2014). 2-aminoethoxydiphenyl borate (2-APB), an inhibitor of the IP_3_ receptor, interacts with several TRP channels. It activates the warm sensitive TRPV1-3, and inhibits several other members of the family, including TRPM3 (Xu et al., 2005). The non-steroidal anti-inflammatory fenamates also inhibited multiple TRP channels, but only mefenamic acid was found to be selective for TRPM3 (Klose et al., 2011). The phospholipase C inhibitor compound U73122 is also suggested to inhibit TRPM3 activation (Leitner et al., 2016).

TRPM3 is inhibited by ononetin, a deoxybenzoin from the plant Ononis spinosa (spiny restharrow), which belongs to the *Fabaceae*. The channel is also blocked by the citrus fruit flavanones hesperetin, naringenin, eriodictyol, liquiritigenin, and isosakuranetin, among which isosakuranetin is the most potent blocker reported until today (Straub et al., 2013a,b). Isosakuranetin and related compounds were also shown to inhibit acute thermal nociception (Straub et al., 2013a) and neuropathic pain (Jia et al., 2017). Another non-steroidal anti-inflammatory drug, diclofenac was also characterized as a TRPM3 blocker, inhibiting agonist-induced currents (Suzuki et al., 2016). The anticonvulsant primidone and the tetracyclic antidepressant maprotiline are also effective blockers of TRPM3. Primidone was found to block the main pore currents evoked by heat, Nifedipine, and PregS, as well as the alternative pore current induced by PregS and clotrimazole. Primidone also attenuated TRPM3-mediated acute thermal pain and heat hyperalgesia. Importantly the IC_50_ value of primidone is in the range of its therapeutic plasma concentrations (Krügel et al., 2017). Recently, we demonstrated that volatile anesthetics can also inhibit TRPM3 in slightly higher concentrations than reached in the plasma during general anesthesia (Kelemen et al., 2020). Although antidepressants, anticonvulsants and volatile anesthetics probably target ion channels in the central nervous system, the putative role of TRPM3 in their therapeutic effect remains to be elucidated.

Interestingly, primidone and volatile anesthetics inhibited both the classical and the non-canonical pore mediated currents, which suggests that these inhibitors do not act as classical pore-blockers but rather inhibit a more general conformational change in the proteins (Krügel et al., 2017; Kelemen et al., 2020).

## Role of TRPM3 in the Brain

### Expression of TRPM3 in Various Brain Regions

The brain represents, next to the kidney, one of the tissues with the highest indicated TRPM3 expression. High levels of TRPM3 mRNA were found in several studies of whole brain tissues from rodent and human (Fantozzi et al., 2003; Grimm et al., 2003; Lee et al., 2003; Oberwinkler et al., 2005; Fonfria et al., 2006; Inoue et al., 2006; Wagner et al., 2008; Gilliam and Wensel, 2011; Jang et al., 2012). More detailed expression analysis showed a high abundance of TRPM3 in the choroid plexus, the cerebellum, the forebrain and the hippocampus (dentate gyrus), among others (Lee et al., 2003; Oberwinkler et al., 2005; Kunert-Keil et al., 2006; Hasselblatt et al., 2009; Hoffmann et al., 2010; Zamudio-Bulcock et al., 2011; Oberwinkler and Philipp, 2014) (Table 1). Likewise, TRPM3 also displays a diverse distribution pattern over several different cell types within the brain. TRPM3 expression was found in neuronal cells, epithelial cells as well as in oligodendrocytes (Hasselblatt et al., 2009; Hoffmann et al., 2010; Zamudio-Bulcock et al., 2011), but on a functional level it was only confirmed in cerebellar Purkinje neurons (Zamudio-Bulcock et al., 2011) and in oligodendrocytes isolated from whole brain tissue (Hoffmann et al., 2010) (Table 1). Actually, to date no structured analysis of the TRPM3 expression was carried out in the different brain areas and the variety of used detection techniques makes it almost impossible to compare the TRPM3 expression between different brain regions (Oberwinkler and Philipp, 2014).

**Table 1:** Overview of reported brain regions expressing TRPM3.

**Brain region**	**Cell type**	**Tested expression level**
Whole brain	Not specified (n.s.) Oligodendrocytes	mRNA Protein mRNA Protein Functional
Basal ganglia	n.s.	mRNA
*Substantia nigra*	n.s.	mRNA
Brain stem	Neuronal cells	Protein
	Oligodendrocytes	Protein
Cerebrum	n.s.	mRNA
Corpus callosum	Oligodendrocytes	Protein
Choroid plexus	n.s.	mRNA
	Epithelial cells	mRNA
Cerebellum	n.s.	mRNA
		Protein
	Purkinje cell	Protein
		Functional
Cortex	Neuronal cells	protein
Fimbria hippocampi	Oligodendrocytes	protein
Forebrain	n.s.	mRNA
Hippocampus	n.s.	mRNA
*Dentate gyrus*	n.s.	mRNA
Hypothalamus	n.s.	mRNA
Locus coeruleus	n.s.	mRNA
Tenia tecta	n.s.	mRNA
-	Oli-neu/OLN-93 cells	Protein Functional

*Brain regions, cell types and the levels of the reported expression for TRPM3 are summarized; n.s., not specified*.

It is interesting to note that several different splice variants of TRPM3 were described to be expressed in the mouse brain, with a total of seven alpha (Oberwinkler et al., 2005; Frühwald et al., 2012) and 17 beta variants (Frühwald et al., 2012; Oberwinkler and Philipp, 2014). Although it was reported that certain splice variant mRNA levels are tissue- and development-dependent in the brain (Hoffmann et al., 2010; Held et al., 2020), it remains unknown whether these different splice variants are functionally expressed in the reported tissues and whether they may be involved in mechanisms regulating TRPM3 activity in a cell-specific manner.

### Function of TRPM3 in the Brain

Since TRPM3 is a non-selective, calcium-permeable cation channel, TRPM3 activity will result in a depolarization of the neuronal membrane. So far, the only studies that showed functional TRPM3 in brain cells were performed in primary oligodendrocytes isolated from the whole brain (Hoffmann et al., 2010) and in cerebellar Purkinje neurons in brain slices (Zamudio-Bulcock et al., 2011). Furthermore, a functional role of TRPM3 in the choroid plexus was suggested (Millar and Brown, 2006; Millar et al., 2007). These studies provided molecular evidence of TRPM3 activity, using allegedly TRPM3-specific pharmacology or a dominant-negative TRPM3 protein block. Despite this molecular evidence, no further efforts have been made to investigate potential effects of TRPM3 activation on a behavioral or (patho)physiological level in these or other studies. Given the expression of TRPM3 in the brain areas mentioned above, certain assumptions can be made concerning the potential physiological roles of TRPM3 in the brain. For instance, the high mRNA expression and functional activity of TRPM3 in cerebellar Purkinje neurons suggests a role of TRPM3 in the coordination of movement (Beckstead, 1996; Purves, 2004). Furthermore, the high expression of TRPM3 in the choroid plexus (Oberwinkler et al., 2005; Millar and Brown, 2006; Millar et al., 2007), may indicate a potential role of TRPM3 in the ion homoeostasis that is necessary for the production of the cerebrospinal fluid (Damkier et al., 2013). Moreover, high levels of TRPM3 in the hippocampus (Oberwinkler et al., 2005; Kunert-Keil et al., 2006; Hoffmann et al., 2010) hint at a potential role in memory formation and consolidation (Andersen, 2007). Fittingly, some endogenous TRPM3 (ant)agonists are known to influence synaptic signaling and memory functions. For instance, the endogenous TRPM3 activator PregS was reported to increase long-term potentiation at hippocampal CA1 synapses (Sabeti et al., 2007), which may lead to memory-improving effects (Dastgheib et al., 2015). Although these actions of PregS were often attributed to effects on NMDA and GABA_A_ receptors (Paul and Purdy, 1992; Mayo et al., 1993; Akk et al., 2001; Horak, 2004), it is equally plausible that effects of PregS were partially mediated via TRPM3 channel activation (Wagner et al., 2008). Similarly, estradiol, a reported TRPM3 antagonist, was shown to enhance memory consolidation mediated via the dorsal hippocampus (Tuscher et al., 2019). In addition, three recent studies reported in parallel on the modulation of TRPM3 by G_βγ_-proteins via a signaling cascade with GPCRs, such as GABA_B_-, μ-opioid- and NPY receptors (Badheka et al., 2017; Dembla et al., 2017; Quallo et al., 2017). Of note, all of these receptors can be found abundantly in the brain (Hill and Bowery, 1981; Delfs et al., 1994; Reichmann and Holzer, 2016), and the inhibitory effects of endogenous, brain-relevant GPCR ligands such as somatostatin (Martel et al., 2012) and morphine (Beltrán-Campos et al., 2015) on TRPM3 currents were illustrated in these studies (Badheka et al., 2017; Dembla et al., 2017; Quallo et al., 2017). However, all of these studies were performed in heterologous expression systems or peripheral sensory neurons and no brain tissue was used to confirm similar actions. Nevertheless, it can be hypothesized that these mechanisms are not exclusive to peripheral nerves, and that receptor-mediated modulation of TRPM3 may also occur in brain tissue. Undoubtedly, the identification of several brain-relevant receptors and ligands that are either directly or indirectly modulating TRPM3 strengthens the evidence that TRPM3 might present an important player in various brain functions. However, such assumptions still have to be confirmed in experiments specifically designed to investigate the here hypothesized or other functions.

### TRPM3 in Brain Disorders

The first link between TRPM3 and brain pathologies was reported in 2009, when Kuniba et al. (2009) performed molecular karyotyping in 17 patients and mutation screening in 41 patients with Kabuki syndrome (KS), a multiple congenital anomaly/mental retardation syndrome (MCA/MR). They identified a chromosomal region that also contains the *TRPM3* gene as a potentially contributing factor in the manifestation of KS. A few years later, Pagnamenta et al. reported a rare *TRPM3* exon deletion in a family with autism and proposed this deletion to contribute to the autism phenotype that was observed in these patients (Pagnamenta et al., 2011). Additionally, TRPM3 overexpression was observed in benign and malignant choroid plexus tumors (Hasselblatt et al., 2009; Japp et al., 2015, 2016). Very recently, TRPM3 was suggested to be involved in mood and anxiety disorders, as an interesting potential player in post-partum mood disorders (Thippeswamy and Davies, 2020). This suggestion was based on the facts that the PregS level-regulating steroid sulfatase (STS) enzyme deficiency is positively linked to mental health conditions and depression in human patients (Cavenagh et al., 2019) and PregS levels were reportedly increased in STS-deficient patients (Sánchez-Guijo et al., 2016). Fittingly, it was also shown that TRPM3 expression was altered in a mouse model of bipolar disorder due to serotonin depletion (Maddaloni et al., 2018), thereby supporting the idea that TRPM3 may regulate mood conditions. However, no hard evidence was provided yet for the mechanistic involvement of TRPM3 in the development and/or manifestation of the above-studied diseases. Considering that several other genes were found to be altered in these patients, conclusions concerning the role of TRPM3 in the investigated pathologies should be regarded with caution.

Interestingly, more evidence for a role of TRPM3 in brain pathologies was provided by a Ca^2+^ influx assay-based drug screening study that identified the clinically approved and commonly used anticonvulsant drug primidone and the anti-depressant maprotiline as potent and relatively selective TRPM3 inhibitors (Krügel et al., 2017). Both drugs were able to completely block the channel activity with IC_50_ values of ~0.6 μM for primidone and ~1.3 μM for maprotiline. However, despite this promising pharmacological profile of maprotiline, reported plasma concentrations during patient treatment are not reaching the concentration ranges necessary to inhibit TRPM3. In contrast, the plasma concentrations of primidone that are obtained in patients treated for epilepsy are in the range of concentrations needed to induce a full block of the PregS-induced TRPM3 currents in a HEK cell culture model (Krügel et al., 2017). Given that there is no consensus on the mechanism for the anticonvulsant action of primidone, TRPM3 represents a potential novel target of this anti-epileptic drug. It can be speculated that a downregulation of the TRPM3 activity might stabilize neuronal membrane potential and/or decrease presynaptic calcium release, thereby rendering the cells less susceptible to damaging overexcitation.

**Figure 2:**
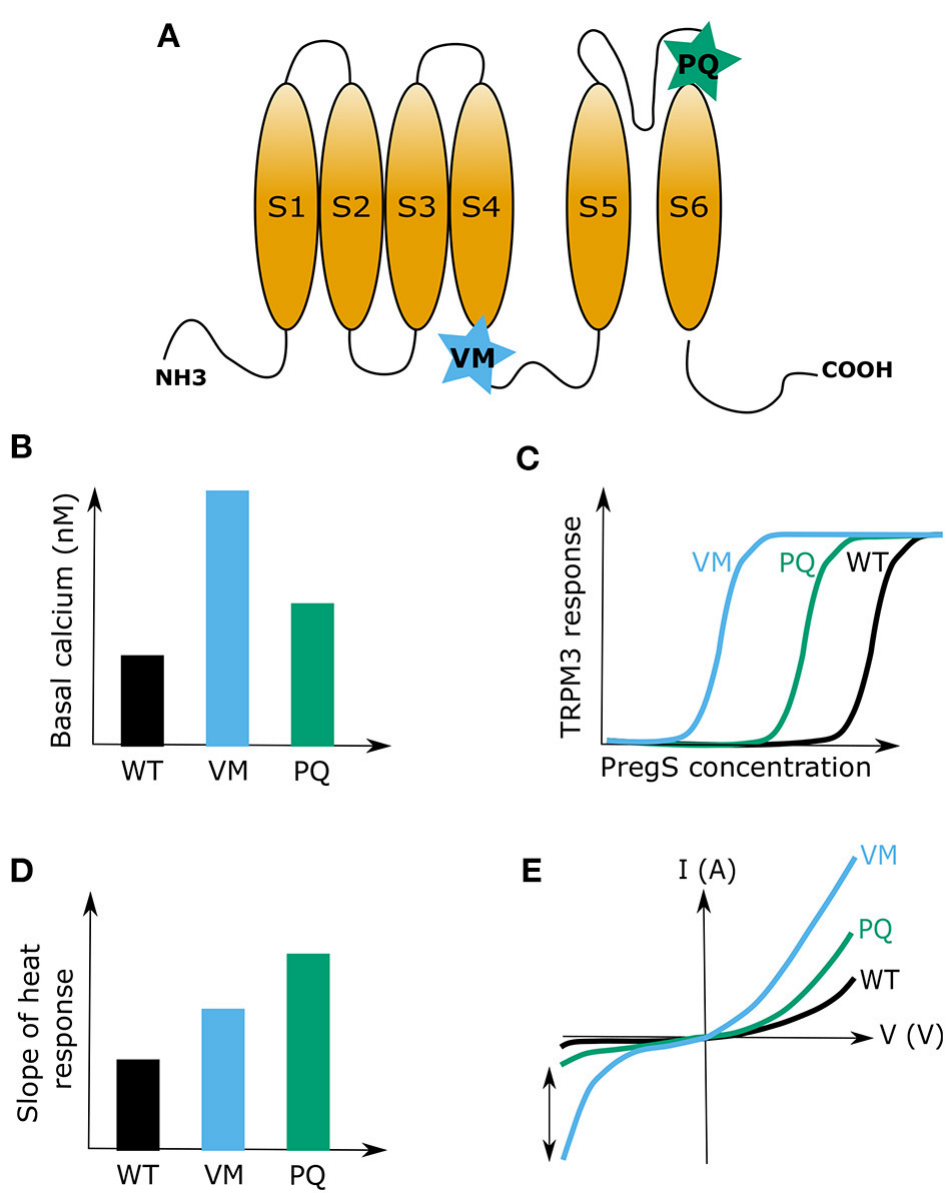
Summary of the two reported developmental and epileptogenic encephalopathy (DEE) disease mutations in TRPM3. **(A)** Cartoon of a TRPM3 channel subunit indicating the positions of the valine to methionine (VM) and proline to glutamine (PQ) substitution mutations. **(B)** Representative graph indicating differences in basal calcium levels between HEK cells that express the TRPM3 wild type channel or the mutant channels VM or PQ. **(C)** Representative graph showing the leftward shift of the concentration-response curve of pregnenolone sulfate (PregS) for the two TRPM3 mutations (VM and PQ) causing developmental and epileptic encephalopathies (DEE). **(D)** Illustration of the heat sensitivity increase for the two DEE mutations compared to wild type (WT) TRPM3. **(E)** Typical examples of current-voltage (I-V) plots for wild type TRPM3 and the DEE mutants VM and PQ during activation with PregS. Note the increase in inward currents for both channel mutants. The two-sided arrow is indicating the high increase in inward currents for the VM mutant, which was attributed to the opening of the alternative ion pore in TRPM3.

Finally, a recent case study reported two *de novo* mutations in the *TRPM3* gene to be the cause of DEE in a total of eight patients between the age of 4 and 38 (Dyment et al., 2019). All eight patients in this study were heterozygous for a *TRPM3* mutation and the majority of them were male (six out of eight). From all eight patients, seven carried an identical point mutation in the linker region between transmembrane segments 4 and 5. This mutation resulted in the substitution of a valine with a methionine (VM mutation) (Figure 2A). The remaining patient carried a proline to glutamine substitution (PQ mutation) at the boundary of the TRPM3 pore-forming loop (Dyment et al., 2019) (Figure 2A). More supporting evidence of these findings was given last year by de Sainte Agathe et al. (2020) who reported about another female DEE patient carrying the VM TRPM3 mutation. Interestingly, this patient did not have epilepsy at the moment of assessment, although it could not be excluded that this might occur in a later stage of life, as was observed in one patient described in Dyment et al. (2019) Although both studies did not further investigate the molecular mechanisms surrounding the disease phenotype, two other research teams performed thorough biophysical characterizations of the two reported TRPM3 disease mutations in *in vitro* cell systems (Van Hoeymissen et al., 2020; Zhao et al., 2020). Both studies concluded that the mutations are causing a gain-of-function in TRPM3, which results in an increased basal channel activity with elevated calcium concentrations at rest, a leftward shift of the concentration-response curve for the endogenous agonist PregS and an increased sensitivity to heat stimuli (Figures 2B–D). Of note, it was shown that the VM mutation additionally results in the opening of the earlier described alternative ion permeation pathway (Vriens et al., 2014a; Held et al., 2015a, 2016) in TRPM3 upon sole application of PregS (Van Hoeymissen et al., 2020). This causes a dramatic increase of the inward currents at physiological resting membrane potentials (Figure 2E). Despite these detailed biophysical characterizations, it remains uncertain how a gain-of-function in the TRPM3 protein causes the observed DEE disease phenotype. Considering the essential role of calcium as a signaling molecule in several neuronal pre- and post-synaptic mechanisms, such as vesicle release, cell depolarization, receptor (de)phosphorylation and internalization as well as in the expressional regulation of proteins (Beattie et al., 2000; Brini et al., 2014), it is not unlikely that the recently studied TRPM3 DEE mutants may have detrimental effects in disease-carrying patients. High calcium levels in the pre- or post-synaptic site of excitatory synapses, which can be caused by an elevated basal channel activity (Figure 2B) or an increased neurosteroid-induced activity (PregS) (Figures 2C,E), might lead to an elevated firing frequency of excitatory post-synaptic neurons. This may subsequently result in hyper-excitable neuronal cells and explain the observed epileptic phenotype (Badawy et al., 2009). Moreover, the patients show initial developmental deficits prior to the epileptic phenotype (Dyment et al., 2019; de Sainte Agathe et al., 2020). It is hard to predict the exact mechanisms that lead to such developmental abnormalities, but it can be speculated that high calcium levels in mutant TRPM3-expressing brain cells may cause neuronal cell death (Toescu, 1998) or that mutated TRPM3 is abundantly expressed in certain inhibitory neurons, which could lead to abnormal synaptic plasticity (Baroncelli et al., 2011). It is definitely intriguing that the sensitivity of TRPM3 to hormonal clues is increased in the TRPM3 DEE mutant channels (Figures 2C,E) (Van Hoeymissen et al., 2020; Zhao et al., 2020), as hormones are known to be centrally involved in developmental regulations (McEwen, 1988, 1992). However, at this point, our knowledge regarding the functional roles of TRPM3 in the brain is very confined, and any kind of mechanistic speculation is rather premature. To illuminate the disease-causing processes in the brain, it first seems necessary to understand, which exact brain areas are affected in the disease-carrying patients, and subsequently confirm and determine specific TRPM3 functions in the various cell types of these brain regions. Clearly, a genetic animal disease-model would be of high scientific value to address such questions, and to shed light on the disease-causing mechanisms of TRPM3 mutations in the brain.

## Discussion

Several lines of evidence suggest TRPM3 as an emerging interesting novel player in brain physiology and pathology. First, TRPM3 was shown to be abundantly expressed and functionally active in different brain regions (Oberwinkler and Philipp, 2014). Furthermore, TRPM3 can be modulated by several endogenous brain ligands and receptors (Held et al., 2015b; Badheka et al., 2017; Csanády, 2017; Dembla et al., 2017; Quallo et al., 2017). In addition, TRPM3 was also shown to be targeted by a commonly used anti-convulsion drug, primidone (Krügel et al., 2017), a compound of which the exact molecular actions are up until today still illusive. However, a brain-specific interaction of these drugs and receptors with TRPM3, and the resulting consequences for brain functions still have to be demonstrated. Finally, various genetic alterations in the *TRPM3* gene were linked to several neurological disorders in human patients (Kuniba et al., 2009; Pagnamenta et al., 2011; Japp et al., 2016; Dyment et al., 2019; de Sainte Agathe et al., 2020) (Table 2). It is interesting to note that almost all of the neuropathologies that were linked to the *TRPM3* gene resulted in a state of intellectual disability in the affected patients. This may suggest a vital role of TRPM3 in neuronal development and could indicate that TRPM3 is of particular importance in defined brain regions. However, so far there are no studies that investigated genetic *Trpm3* alterations in a systematic way, by linking molecular TRPM3 functions directly to the *in vivo* phenotypes that are caused by these alterations. Clearly, there is an urgent need for more detailed functional studies of TRPM3 in the brain.

**Table 2:** Overview of reported diseases that were linked with *TRPM3* alterations.

**Disease**	**TRPM3 related changes**	**References**
Autism	Deletion of exons 1-9 of *TRPM3*	Pagnamenta et al., 2011
Choroid plexus tumors	Up-regulation of *TRPM3* expression	Hasselblatt et al., 2009; Japp et al., 2015, 2016
Developmental and epileptic encephalopathies (DEE)	*De novo* substitutions in TRPM3 (V837M and P937Q)	Dyment et al., 2019; de Sainte Agathe et al., 2020
Kabuki syndrome	Deletion of chromosomal region that encodes *TRPM3* (9q21.11-q21.12)	Kuniba et al., 2009

*For each disease the reported gene or protein alterations of TRPM3 are presented together with the according case studies*.

As TRPM3 represents a non-selective, cation permeable ion channel with a high permeability for calcium, it is very likely that TRPM3 has an impact on neuronal functions and development by affecting electrical and chemical signals in brain regions where it is expressed. Therefore, thorough morphological, molecular and electrophysiological assessments of different brain areas, circuits, and cells that were shown to express TRPM3 RNA or protein, are warranted in the future in healthy and diseased brains. Additionally, behavioral screening experiments to specifically address selected brain functions linked to the investigated areas, in combination with (brain region-specific) pharmacological targeting or genetic alterations in animal models would be of immense value for our future quest to explore the role(s) of TRPM3 in the brain. Considering the broad hormonal regulation profile of TRPM3 (as discussed in section TRPM3 Is a Steroid Regulated Channel), it would be of further scientific value to apply behavioral tests to animals of different gender and different developmental stages. Such experiments will not only provide insights into the physiological roles of TRPM3 in the brain but could also illuminate the mechanisms of disease in selected TRPM3 animal disease models.

Obviously, more research is required to investigate TRPM3 expression, regulation and function in different brain regions and cell types, and to validate its role in brain (patho)physiology. Nevertheless, given our current knowledge about the molecular and biophysical properties of TRPM3 and its recent genetic links to brain pathologies, it is tempting to imagine TRPM3 as an attractive potential new target for future drug interventions in neurological diseases such as epilepsy or autism spectrum disorders.

## Author Contributions

KH and BIT drafted, corrected, and wrote this article. BIT was responsible for the funding acquisition. All listed authors qualify for authorship and all authors qualifying for authorship are listed above.

## Conflict of Interest

The authors declare that the research was conducted in the absence of any commercial or financial relationships that could be construed as a potential conflict of interest.

## Abbreviations

2-APB: 2-aminoethoxydiphenyl borate
CA1: Cornu Ammonis 1
DEE: developmental and epileptic encephalopathies
DHEA: dehydroepiandrosterone
DRG: dorsal root ganglia
GABA: γ-aminobutyric acid
Gβγ: G beta-gamma complex
GIRK: G-protein-coupled inward rectifier potassium channel
ICFR: *r*egion *i*ndispensable for *c*hannel *f* unctions in TRPM3
*I-V* plot: current-voltage plot
KS: Kabuki syndrome
K_v_: voltage-gated potassium channel
MCA/MR: multiple congenital anomaly/mental retardation syndrome
MITF: microphthalmia/melanogenesis-associated transcription factor
N.at-K_v_3.2: flatworm voltage-gated potassium channel 3
Na_v_: voltage-gated sodium channel
NFATc1: nuclear factor of activated T cells type c1
NMDA: N-methyl-D-aspartate
Pax6: paired-box 6 transcription factor
PD: pore domain
PtdIns(4,5)P_2_: phosphatidylinositol 4,5-bisphosphate
PtdIns(3,4,5)P_3_: phosphatidylinositol 3,4,5-trisphosphate
PregS: pregnenolone sulfate
RANKL: receptor activator of nuclear factor kappa-B ligand
S1-6: transmembrane segments 1-6
STS: steroid sulfatase
TRPM: transient receptor potential channel melastatin
TRPC: transient receptor potential channel canonical
TRPV: transient receptor potential channel vanilloid
VM/PQ mutation: human TRPM3 DEE point mutations with amino acid substitutions valine to methionine (VM) and proline to glutamine (PQ)
VSD: voltage-sensing domain
WT: wild type.
